# Boolean modelling as a logic-based dynamic approach in systems medicine

**DOI:** 10.1016/j.csbj.2022.06.035

**Published:** 2022-06-17

**Authors:** Ahmed Abdelmonem Hemedan, Anna Niarakis, Reinhard Schneider, Marek Ostaszewski

**Affiliations:** a Luxembourg Centre for Systems Biomedicine, University of Luxembourg, Esch-sur-Alzette, Luxembourg; b Université Paris-Saclay, Laboratoire Européen de Recherche pour la Polyarthrite rhumatoïde – Genhotel, Univ Evry, Evry, France; c Lifeware Group, Inria, Saclay-île de France, 91120 Palaiseau, France

**Keywords:** Logical modelling, Boolean networks, Modelling formats, Systems Biology standards, BN, Boolean Network, BF, Boolean Function

## Abstract

Molecular mechanisms of health and disease are often represented as systems biology diagrams, and the coverage of such representation constantly increases. These static diagrams can be transformed into dynamic models, allowing for *in silico* simulations and predictions. Boolean modelling is an approach based on an abstract representation of the system. It emphasises the qualitative modelling of biological systems in which each biomolecule can take two possible values: zero for absent or inactive, one for present or active. Because of this approximation, Boolean modelling is applicable to large diagrams, allowing to capture their dynamic properties. We review Boolean models of disease mechanisms and compare a range of methods and tools used for analysis processes. We explain the methodology of Boolean analysis focusing on its application in disease modelling. Finally, we discuss its practical application in analysing signal transduction and gene regulatory pathways in health and disease.

## Graphical abstract

**Figure:**
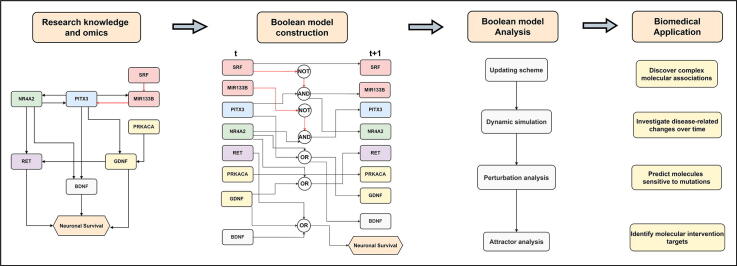

## 1 Introduction

Extensive amounts of omics data generated to understand disease mechanisms require interpretation to formulate meaningful hypotheses [1]. Pathway databases [2], [3], [4] give an overview of disease-related processes, while mathematical models give qualitative and quantitative insights into their complexity. Similarly to pathway databases, mathematical models are stored and shared using dedicated platforms [5], [6], [7], [8], [9]. Moreover, community-driven initiatives such as disease maps [10] encode disease-specific mechanisms in both computable and diagrammatic form using dedicated tools for diagram biocuration [11], [12], [13] and visualisation [14], [15]. In all cases, computationally readable content can be used as a scaffold to build dynamic models in an automated fashion to investigate the dynamic properties of the system [16].

Modelling of a biological process depends on the scope and it may vary depending on the nature of the process (e.g.signalling vs metabolic). The experimental design and resulting data also influence the model structure and analysis [17]. Dynamic modelling approaches include Boolean or Multi-valued Networks [18], Petri nets [19] or Ordinary Differential equations (ODEs) [20]. However, model parameterisation is a challenging task [21] making logical models an interesting alternative [22], [23]. Boolean models are qualitative rather than quantitative and do not require detailed kinetic information. However, in some research areas, such as pharmacogenomics, presenting data to simple Boolean models may be challenging, and does not introduce the best description of the biological system [24]. Therefore, researchers studied the qualitative nature of Boolean models, facilitating the integration with other quantitative methods to allow better analysis [25], [26], [27]. Such methods, including ODEs and Petri nets, combined with BNs and constraint-based models, show that Boolean models are useful scaffolds for quantitative models [24].

Boolean network (BN) represents a dynamic system under the Boolean formalism, where the state of biomolecules has two possible values, one or zero, and changes following their interactions described by Boolean functions (BFs). BFs define the state of the outputs based on the interaction inputs and their internal logic. The order of evaluating BFs in a BN is governed by an updating scheme [28]. This straightforward framework of BNs was applied to a range of biological problems [29], [30], [31] and model qualitative behaviours [32], [33]. Here, we review the application of Boolean modelling to systems medicine problems. First, we explain the modelling process itself, and follow by reviewing applications of Boolean modelling in clinical and translational medicine. We conclude by discussing emerging tools and methods improving the reproducibility and reuse of such models in biomedical research.

## 2 Boolean modelling process

Boolean models represent a logical formalism, where available variables have binary values one (ON) or zero (OFF). These variables are connected by BFs, which define the state of output variables based on input variables. BFs (interactions) connect the variables (nodes) in a model, creating a Boolean Network (BN). The updating schemes define conditions and order in which the BFs are calculated.

A BN can describe the dynamics of a biological system, where biomolecules are represented by the variables and BFs encode the interactions between the biomolecules, describing the behaviour of regulated outputs based on regulator inputs. Connectivity and logic of BNs are usually constructed based on biocuration, by defining relevant biomolecules and interactions based on available literature. BNs can also be built from mechanistic data, like time series or phosphoproteomics [28], [34]. Finally, the updating schemes of BNs need to be selected, to govern the transitions of BN components from one state to another following the defined BFs.

### 2.1 Building the model structure

BNs can be manually built from literature. This requires selection of key components (nodes) to represent the biological system, including non-physical elements such as pathway endpoints (phenotypes). The interactions (edges) between these network components are based on relevant literature and represent logical dependencies of the components, i.e. the BFs of the system [35]. When building a BN, it is crucial to define its purpose and use cases to better determine information that should be collected and kept throughout the analysis [36]. Importantly, a process of manual curation depends on the expertise and reasoning of the curator. To ensure reliability of such models, curated BNs should be well documented, harmonised with community guidelines, and reproducible with the corresponding data [37]. The literature based curation can be complemented by model inference from time-series data, which allows constructing hypothesis-free models [38], [39], [40], [41].

For gene expression time series, the data is binarised based on a given threshold to infer ON/OFF states of particular genes in each time step. There are a range of possible approaches for determining an optimal threshold [38], [42], and they should be chosen based on the nature of the measured biological process. After binarization, BFs can be inferred based on the sequence of state changes of selected genes, which may involve adjustments of the original binarization thresholds [43], [44]. Importantly, such inference may be inaccurate for coexpressed genes [43]. To improve accuracy, different binarization methods can be combined to reduce the network complexity and rank common candidates by the performance of inferred models [45]. Another approach to infer the BN from expression data is to first construct a regulatory network and then assign BFs, avoiding data binarization. Here, the TIGRESS algorithm [40] can be used to infer the network and pass it to the TaBooN workflow [41] that infers the fittest BFs based on the compatibility with given expression profiles by using a Tabu metaheuristic[46].

BNs built from literature or inferred from expression data may require further refinement. Such refinement is possible by matching the performance of a BN against additional datasets [29], like perturbation experiments or phosphoproteomic readouts. Based on such data, a BN can be reduced to a version that best explains the validation data. This search can get computationally expensive, requiring heuristic-based approaches to identify a set of the most fitting BNs. For instance, in [47] the algorithm weights the fit between the data and a BN by measuring the deviation between the model steady states and the perturbation data in matched conditions (knockouts, overexpressions). However, this approach is not scalable in large complex networks. To solve this problem, caspo workflows [48] were proposed to infer all optimised BNs by using the Answer Set Programming (ASP) [49]. This approach was used to infer BNs of different cell lines from curated networks and infer their BFs based on phosphoproteomic datasets [48]. Importantly, for each perturbation condition only the corresponding part of the BN is analysed, selected based on the downstream phenotype. The ASP then identifies a set of BNs which fit the experimental data and satisfy a specific condition.

### 2.2 Construction of Boolean functions

**Fig. 1:**
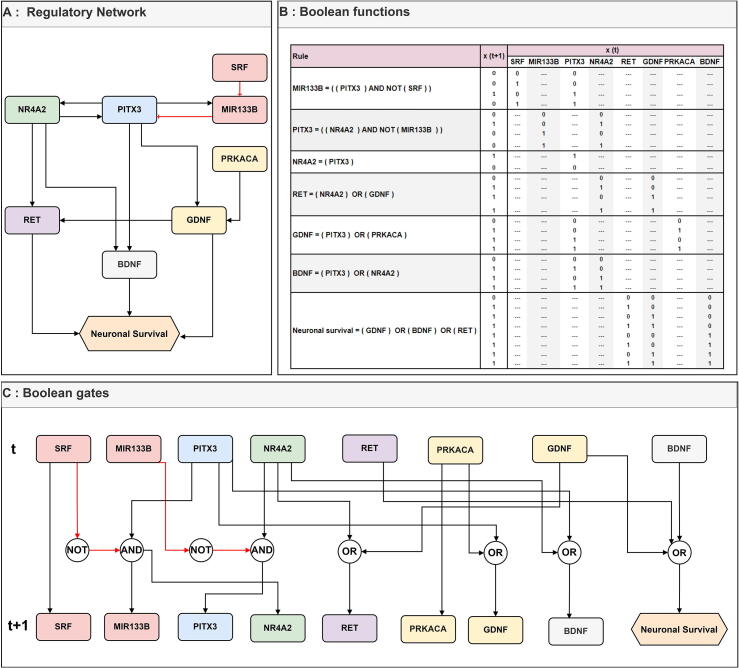
(A) illustrates a simple directed Network [35], with typically used logical functions. Red arrow refers to the inhibition effects. Black arrows refer to the activation effect. (B) shows Boolean functions either in basic logical expressions or as a truth table. (C) shows the Boolean gates AND/OR/NOT, describing the dynamics update from time (t) to (t + 1). (For interpretation of the references to colour in this figure legend, the reader is referred to the web version of this article.)

Biological processes can be modelled as interactions of biomolecules, controlled by their regulators, where BFs describe the relationship between all interaction participants (see Fig. 1). The state of a biomolecule is changed based on these BFs in an iterative manner [50]. In each iteration, the states of biomolecules are changed based on a chosen updating scheme [28].

The three basic BFs are AND, OR, and NOT. BFS can be represented using a truth table in which each row represents a combination of Boolean variables and their output values (see Fig. 1). Other types of BFs are not as widely used, as they describe complex and non-intuitive relationships [51]. One of such functions is a canalysing function, allowing to define a hierarchical relationship between multiple input variables of a BF [52]. A canalysing function has a defined structure with at least one input and a fixed output. An input takes a specific value and determines the value of the function, making the network stable [52].

BFs can also be constructed by probability distributions [51] to represent combinatorial effects of regulations in a simple and interpretable representation [53]. In models with such BFs, called threshold Boolean Networks (TBNs), biomolecule regulation is an additive process, in which the operator functions describe the sequence of events in the regulatory system [32], [54]. The dynamics of a TBN can be described by:

(1) $$x_{i}\left(t+1\right)=\left\{\;\begin{array}{c} 1,\;\;\;\;\;\;\sum_{j}^{\;\;}a_{\mathrm{ij}}\;x_{j\;}\left(t\right)>\;0\\ 0,\;\;\;\;\;\;\;\sum_{j}^{\;\;}a_{\mathrm{ij}}\;x_{j\;}\left(t\right)<\;0\\ x_{i}\left(t\right),\;\;\sum_{j}^{\;\;}a_{\mathrm{ij}}\;x_{j\;}\left(t\right)=\;0 \end{array}\right)$$

x i (t+1) represents the expression of the regulated biomolecule i at the next time.

(t+1), and the interaction coefficient a ij refers to the strength and type of regulation that biomolecule j exerts on i. Positive regulation is specified by positive values of and negative regulation by negative values of a ij. Any regulation is a product of the regulator’s state x j (t) and the type and strength of the regulation a ij. The next state of a biomolecule depends on its regulator's state. In particular, the next state x i (t+1) of a biomolecule i is ON if the sum of its regulators' regulatory effects surpasses 0, OFF if the sum is below 0, and when the sum is 0, the state remains the same.

## 3 Analysis of Boolean models

The dynamics of a model are simulated by incremental execution of one or more of its BFs. They change the states corresponding biomolecules in a series of discrete time steps called transitions. The spectrum of all possible transitions is illustrated using the state transition graph. The vertices represent 2^n^ possible states (n: number of the network elements) and the edges represent the transition from a state (s) to another (s′) as follows::

(2) $$T\left(s,s^{\prime }\right)=\;{⋀}_{i=1}^{n}({x\prime }_{i}\;\;\;↔\;\;f_{i}\;\left(x_{i1},x_{i2}...x_{\mathrm{ik}}\right)$$

In which $f_{i}$ is the updating function and $(x_{i1},x_{i2},...x_{\mathrm{ik}}$) are state variables.

**Fig. 2:**
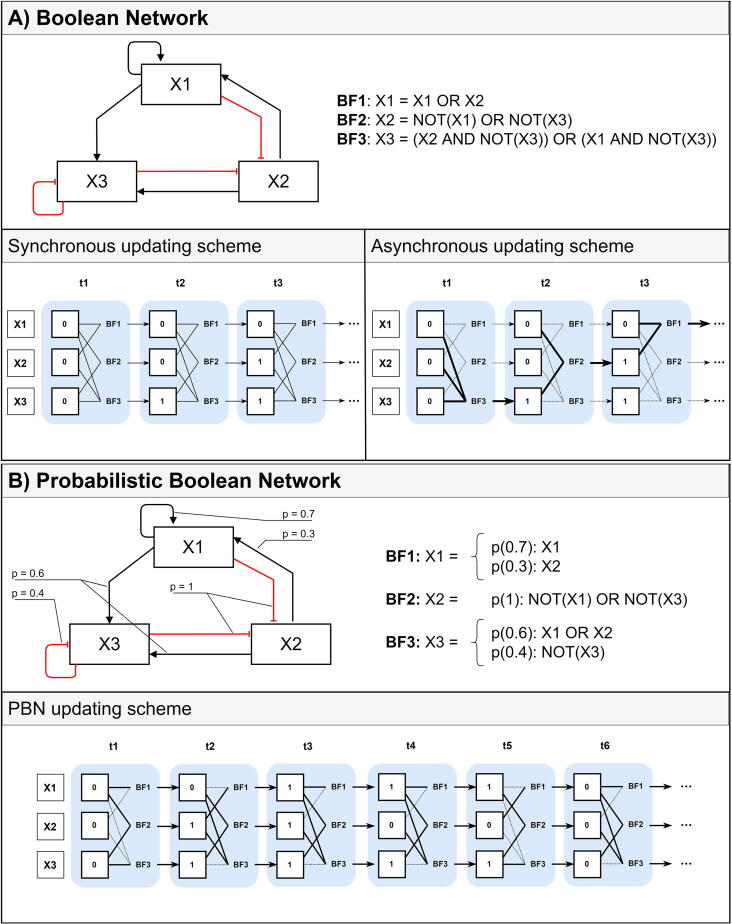
represents the network updating in time for a simple regulatory graph. (A) Boolean network includes three components X1, X2, X3 which have states (zero/one). The dynamics of a component is represented by Boolean function BF. Synchronous updating scheme updates all states at the same time, the successor states have two possible values, one (ON) or zero (OFF). In the asynchronous updating scheme, the start states are not updated at the same time (one state is updated per iteration), the successor states have two possible values one (ON) or zero (OFF). (B) A Probabilistic Boolean network shows that states are updated at the same time and the successor states present different probabilities; p represents the updated probability values of the variables. Importantly, an asynchronous updating scheme can be used in PBNs as well.

The order at which BFs are executed is governed by an updating scheme [55]. The most frequently used are synchronous, asynchronous and hybrid updating schemes [35]. The synchronous scheme updates the state of all biomolecules at the same time (see Fig. 2.A) and is deterministic in nature. Another class is the asynchronous scheme, which updates randomly the state of a single biomolecule per transition (see Fig. 2.A), making each execution non-deterministic [56]. This makes the runtime considerably longer, especially for complex networks. This limitation is addressed by improved updating schemes such as random order asynchronous [57] and deterministic asynchronous [58] that apply reduction techniques to simplify the BNs. Another limitation of synchronous and asynchronous schemes is that they may inaccurately represent mechanisms that need more than one time step. This limitation is addressed by probabilistic BNs (PBN). This scheme assigns probabilities to the BFs, and biomolecules are updated based on this probability (see Fig. 2B) [35], [53].

In a hybrid updating scheme, using synchronous and asynchronous schemes is possible. The synchronous update can include time delays in interactions between regulators and their final products [35], [38]. This scheme assumes partitioning the network into groups, the variables at each group are synchronously updated, but the update between groups is asynchronous, causing time delays during the executing partitions of a network [35], [59].

### 3.1 Attractor analysis

A simulated model can reach a stable dynamic behaviour, where the states of the biomolecules converge to a stable configuration, called an attractor, which is interpreted as a physiological endpoint [35], [50].

An attractor is a state of a BN with no outgoing edges in the state transition graph. Attractors can be classified as i) stable states (fixed points) which are time invariant, and ii) complex attractors – sets of possible outcomes that can be reached following the synchronous and asynchronous scheme [60]. The set of states within an attractor is called the basin of attraction. It can be interpreted as a set of possible biological scenarios, supporting testable hypotheses [61]. In synchronous and deterministic asynchronous schemes, the system may oscillate regularly when attractors form a limit cycle, and each node has not more than one successor. An example of a limit cycle is the cell cycle in models of a eukaryotic cell [62], [63], [64]. In a stochastic asynchronous scheme, the system may oscillate irregularly due to the random initial condition selection leading to loose attractors. That means the network does not oscillate in a cycle due to the target node having more than one successor. It is challenging to interpret complex attractors with large numbers of steady states that oscillate in an irregular cycle.

To find an attractor, the past states of the model are compared to the updated ones to find recurring patterns. This search process can be exhaustive or heuristic. An exhaustive search starts from all states synchronously until the attractor is reached. This mode is mostly limited to small-size networks [65], although a SAT solver can increase the search speed, identifying the possible attractors in large networks with hundreds of components [66]. In turn, the heuristic search starts with a chosen subset of states to identify the attractor synchronously or asynchronously. The heuristic search performs random transitions, creating network states with a high probability. Then, the algorithm computes the forward reachable sets of the network states. If all sets are similar, an attractor is identified [56].

Identifying an attractor in a complex network is challenging. Many reduction techniques were implemented to simplify the original BFs to include a fewer number of operations [58], [67], [68]. This can be achieved by removing components that do not affect the behaviour of the original BFs. For example, some reduction techniques identify the biomolecules whose state do not change, turning the corresponding BFs into a simpler model [58], [67]. In complex BNs, this technique is followed by removing interactions with one input and output and self-loops [67]. Another approach splits the network into strongly connected components (SCCs) to decrease the model complexity, and the simulations are run for all the SCCs independently [69]. Recently proposed Most Permissive Boolean Network simulations (MPBNs) is a paradigm to perform trajectories sampling and to reach the complete set of attractors faster than the asynchronous search, allowing to run more fine-grained simulations [70].

### 3.2 Topology, perturbation, and controllability analysis

Simulation of BNs to identify their stable states and attractors provides insights into the behaviour of the model. From this point, it is possible to predict meaningful interventions towards desired outcomes by analysing the structure of the model and its response to perturbations. To gain insight into the model structure, it is important to study the topology of a BN which may be a necessary prerequisite for some updating schemes and/or attractor analysis (the ones that need modularisation). Such analysis helps to understand the connectivity of the network components and how they affect phenotypes. This information can improve the understanding of BN dynamics under different updating schemes or attractor analysis [71] by identifying structural cycles in the BN topology. Moreover, this information can be used to define components sensitive against perturbations [72].

**Fig. 3:**
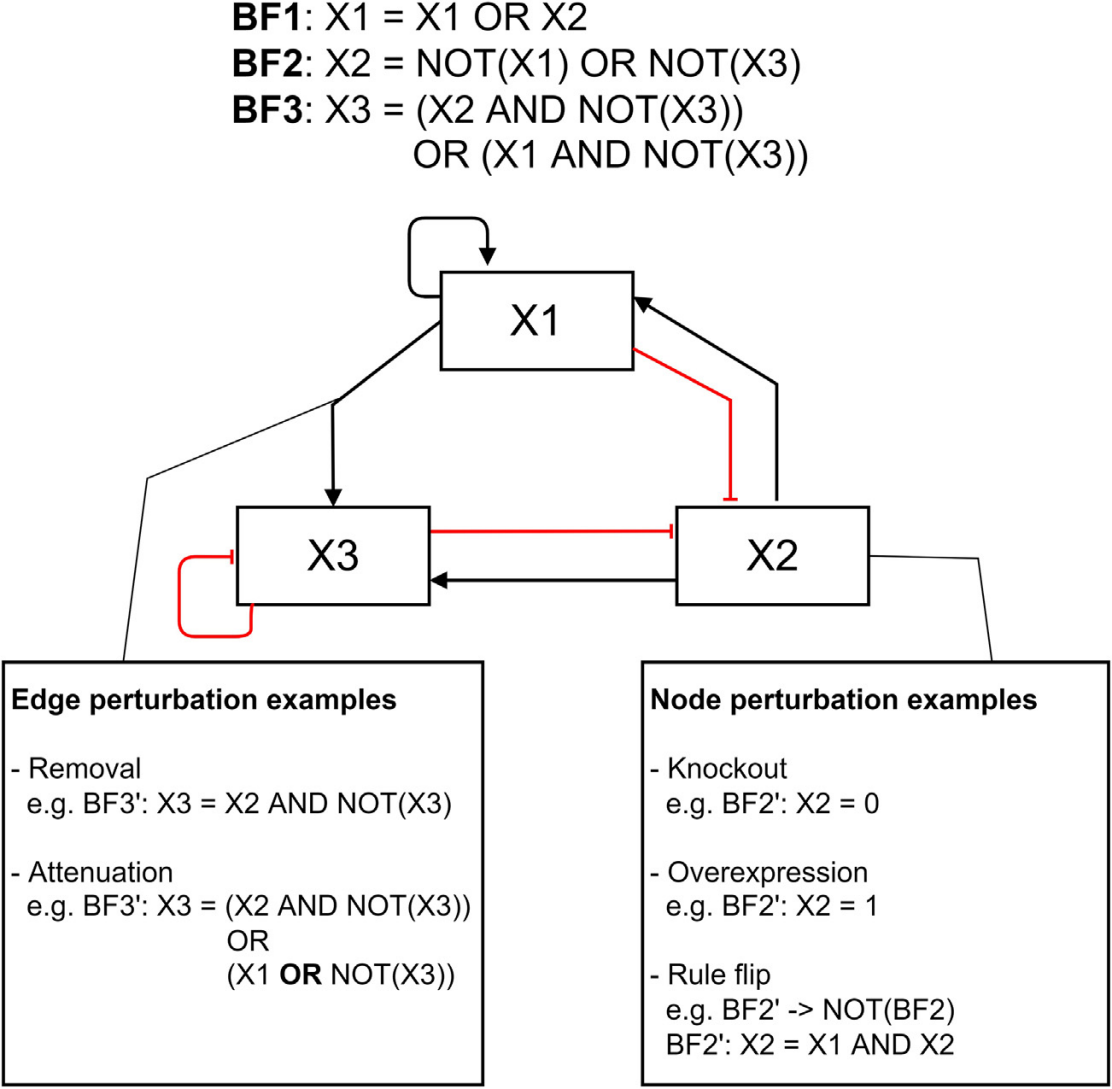
represents a regulatory graph in which the X3 node is subjected to activation (green link)/inhibition effects (red link). Node perturbations represent the changes of the X3 states based on Knockout/overexpression. Edge perturbations represent the changes of functions based on X1 ->X3 interaction mutations. (For interpretation of the references to colour in this figure legend, the reader is referred to the web version of this article.)

Perturbation analysis means changing the state of a biomolecule or its BFs, to analyse the topological robustness and the dynamic resilience of the Boolean model, and the attractors it reaches [73], [74]. Comparing the original attractors with those after perturbation allows evaluating its impact. One of frequently used perturbations sets the state of a biomolecule to a fixed value, zero or one, emulating permanent activation or activation, e.g., due to a drug action. Other types of perturbation may change the rule structure of the BFs, either entirely (rule-flip) or partially. Such partial perturbations are called edge perturbations, as they affect the connectivity of a BN (Fig. 3).

The control of the BNs can be achieved by adding external sets of signals to affect the state of the biomolecules so the model reaches the desirable stable state or attractor [75], [76]. The added signals, represented as additional nodes in the BNs, have no parent interactions and their values are a series of state values corresponding to simulation time steps, guiding the model towards the desirable states. They can represent possible therapies e.g., the control of gene expression essential for therapeutic interventions [75]. The second approach to control a BN is to perturb the states of the network randomly to select the biomolecules that may result in attractors representing the desired outcomes of the model. This approach was implemented as an algorithm [77] that identifies the optimal one-bit perturbation, i.e., the simplest form of perturbation that inverts the states of biomolecules in an attractor, for a given configuration of external inputs.

### 3.3 Boolean modelling formats and tools

**Fig. 4:**
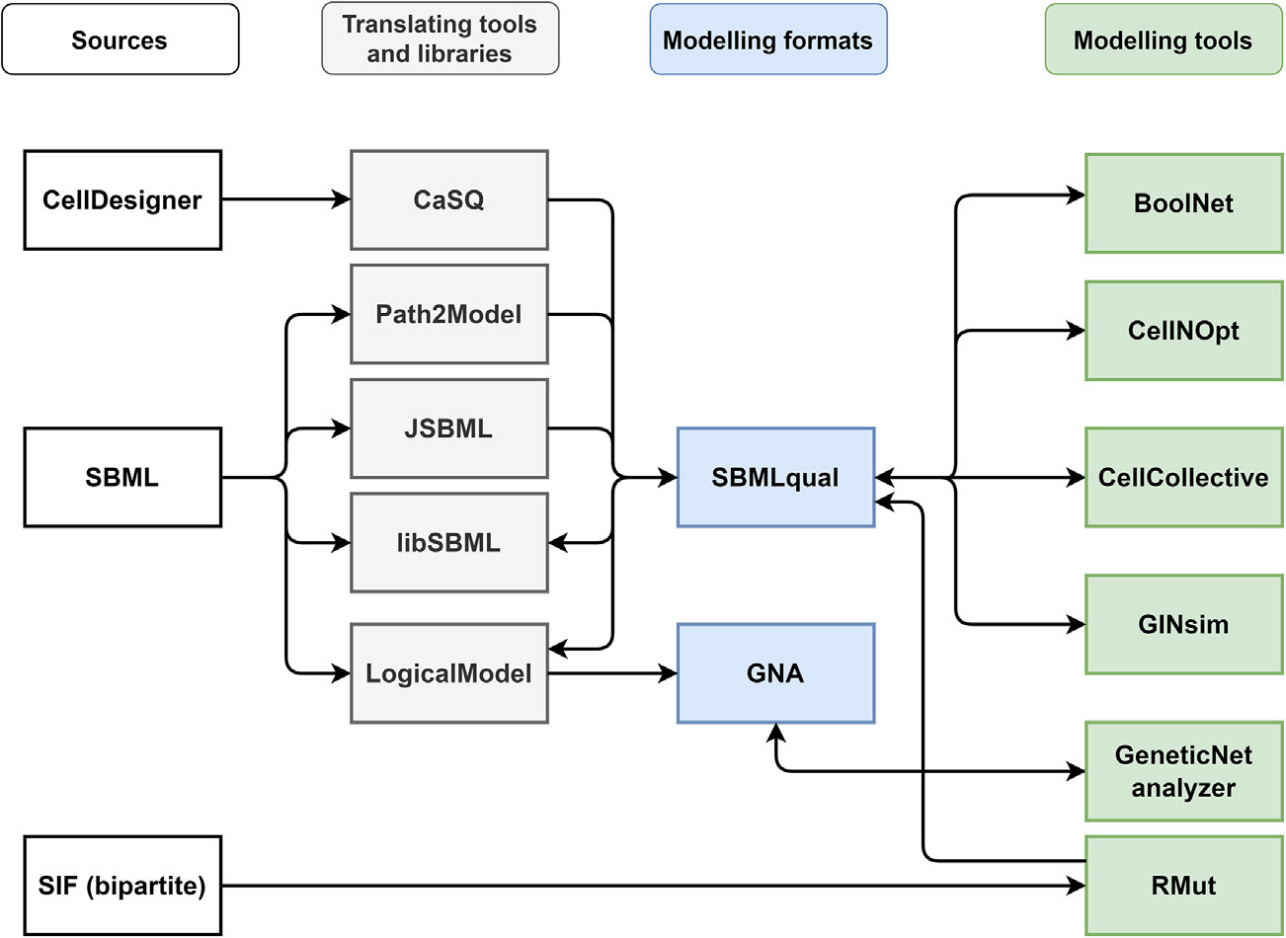
Interoperability of Boolean modelling tools, libraries, and formats. The format of data resources (white colour) can be translated by tools and libraries (grey colour) to modelling formats (blue colour), to be used by the popular Boolean modelling tools (green colour). (For interpretation of the references to colour in this figure legend, the reader is referred to the web version of this article.)

A Boolean model can be constructed and represented using various modelling tools relying on different formats, as illustrated in Fig. 4. One of these formats is the simple interaction format (SIF), which is used for encoding a model topology from a list of interactions, giving an easy solution for combining new interactions to models. SIF is supported by different tools and databases such as Cytoscape [78], OmniPath [79] and Signor [80].

In order to re-use or integrate models, they need to be translated from their original format. Literature-constructed diagrams can be transformed into BNs as a SIF using automated conversion tools (Fig. 4). SIF can be translated into a list of BFs in Boolsim format [81] using a Standardised QUAlitative Dynamic approach (SQUAD) [82]. In addition, GNA allows the encoding of model functions and specifies qualitative values of a model from the experimental literature.[83].

Model annotations can be stored along with the topology and BFs using a SBML-qualitative format (SBML-qual). SBML-qual is a standard format designed by the CoLoMoTo community [84], extending the SBML [85] to represent the qualitative models of biological networks [86]. Pathway diagrams from KEGG, BioCarta and SABIO-RK [87] can be transformed to SBML-qual using PAth2Models [87]. Using a dedicated converter CaSQ [16], CellDesigner SBML format can be translated to SBML-qual. Notably, SBML can be translated into SBML-qual by CellNOpt [29] and SQUAD [82]. However, the SBML-qual format is still incompatible with some tools such as RMut [74], NetDS [88] and CABEAN [89], pointing out that further integration efforts are required to allow reproducibility of SBML-qual models with the incompatible tools.

In the modelling process, a range of tools is available for model inference, simulation, and attractor analysis. In the case of model inference, most represented tools in Table 1 generate Boolean models randomly based on selected information, which is known as random network generation. The two software- CABERNET and caspo- create a Boolean model by augmentation if the topological/ functional characterization is incomplete (Table 1). A user can generate ensembles of models by combining components and interactions with the original network.

**Table 1:** Summary of key tools and their functionalities that were implemented to perform Boolean analysis and simulations. GUI – Graphical User Interface, CL – Command Line.

Tools	Interface	Format	Network generation	Updating scheme	Attractor search	Attractor analysis	Topological analysis
CellCollective [7]	Web, GUI	SBML qual	–	Asynchronous	Heuristic, Exhaustive	–	Centrality
GINSIM [8]	GUI	SBML qual	–	Asynchronous	Heuristic, Exhaustive	–	–
BooleSim [81]	Web, GUI	Own	–	Synchronous	–	–	–
ADAM [90]	Web, GUI	SBML core	–	Asynchronous	Heuristic, Exhaustive	–	–
BoolNet [6], [5]	CL, Cytoscape	SBML qual	Random	Asynchronous	Heuristic, Exhaustive	–	Centrality, Clustering
CellNopt [29]	CL, Cytoscape	SBML core, SBML qual	–	Synchronous	–	–	Centrality
RMut [74]	CL	Own	Random	Synchronous	–	Stability, Controlability	Centrality, Clustering
SQUAD [82]	GUI	SBML core, SBML qual	–	Synchronous	–	–	–
CABERNET [96]	GUI, Cytoscape	SBML core	Random, Augumented	Synchronous	Heuristic, Exhaustive	Stability	Centrality, Clustering
NetDS [88]	GUI	SBML core	Random	Synchronous	Heuristic, Exhaustive	Stability	Centrality
GDSC [95]	Web, GUI	Own	–	Synchronous	Heuristic, Exhaustive	–	Centrality
CANA [92]	CL	Own	Random	Synchronous	Exhaustive	Stability, Controlability	–
CABEAN [89]	CL	Own	–	Asynchronous	Exhaustive	Stability, Controlability	–
ASSA-PBN [93]	CL	Own	Random	Synchronous	Heuristic, Exhaustive	Stability, Controlability	–
caspo [48]	CL	Own	Augumented	Synchronous	–	–	–
BMA [94]	Web, GUI, CL	Own	–	Synchronous	Exhaustive	Stability, Controlability	–

The synchronous updating scheme is a default simulation method, supported by all the reviewed tools. Moreover, CellCollective [7], GINsim [8], ADAM [90], BoolNet, MaBoSS [91] and CABEAN support the asynchronous scheme (Table 1).

Most of the represented tools identify the attractor dynamics with heuristic and exhaustive search except BooleSim [81], CellNopt, RMut and SQUAD. Once the attractors are identified, the stability and controllability check can be performed by RMut, CANA [92], CABEAN, ASSA-PBN [93], BioModel Analyzer BMA [94]. In turn, BoolNet, RMut, GDSC [95] and CABERNET [96] perform the topological analysis of the intrinsic structure of the network.

As mentioned, particular tools such as RMut, NetDS, and CABEAN are incompatible with the SBML-qual modelling format. There may be limitations to the reusability of models created with these tools since they have their own formats. As a result, ensuring interoperability and reproducibility of models is necessary when incompatibilities exist.

## 4 Applications of Boolean modelling in clinical and translational medicine

Boolean modelling was applied in clinical and translational medicine research [50], [97], [98], [99] for various purposes. Simulation of the complex biological systems allowed to predict the activity of pathway endpoints (phenotypes) [100], drug targets [76] and cellular crosstalk [101]. Identifying attractors helped to understand the activity of the phenotypes, since they represent the steady states of biomolecules [102], [103]. Finally, comparing attractors before and after perturbations allowed evaluating the model stability and gave insight into how the in-vivo systems maintain their homeostasis. Below we discuss examples of such applications, as summarised in Table 2.

**Table 2:** selected some of applications of Boolean modelling in clinical and translational medicine.

Models	Size	Type	Reference
T cell signalling (MAPK signalling and PI3K/PKB signalling)	94 nodes/123 interactions	Cell signalling	[104]
TCR signalling, Cytokine signalling, and cell cycle	65 nodes/135 interactions	Cell signalling	[105]
Plasticity of CD4+ T cell differentiation	38 nodes/ 96 interactions	Cell signalling	[106]
TGFB1, IL6, and TNF signalling	38 nodes/ 59 interactions	Cell signalling	[110]
Gastric adenocarcinoma	10 nodes/ 34 interactions	Cancer signalling	[97]
Simplified cancer network	96 nodes/ 249 interactions	Cancer signalling	[112]
RTKs, WNT/β-catenin, TGF-β/Smads, Rb, HIF-1, p53, PI3K/AKT signalling pathways	98 nodes/254 edges	Cancer signalling	[114]
Pro-apoptotic pathways with the growth factor signalling	37 nodes/ 63 interactions	Cancer signalling	[116]
PI3K/AKT1 signalling pathway	30 nodes/ 42 interactions	Cancer signalling	[115]
Signalling pathways around BRAF in colorectal and melanoma cancers.	33 nodes/43 interactions	Cancer signalling	[100]
Signalling in prostate cancer	133 nodes/ 449 interactions	Cancer signalling	[120]

### 4.1 Modelling of cell signalling

A complex signalling network can determine cellular decisions, but the kinetic parameters and quantitative data that enable dynamic modelling may not be sufficient. Therefore, computational approaches based on the qualitative structure of these networks are of great interest. Boolean modelling of cellular signalling provided insights into the process of signals and the interactions between regulators and target molecules. A model of *T cell signalling* included: i) a T cell receptor, its co-receptors CD4/CD8, and CD28 which regulates T cell function, ii) selected *MAPK signalling* and *PI3K/PKB signalling* driving cellular activation and differentiation. The model was able to reproduce the literature and experimental results upon different activation scenarios of TCR, CD4 and CD28. Moreover, it reproduced the T cell phenotype in response to knockouts and predicted unexpected activation of the *PI3K/PKB signalling* pathway after TCR activation [104]. This model was extended [105] into large BNs modelling a regulatory Th cell. *TCR signalling*, *cytokine signalling, and cell cycle* models were studied separately, and integrated into a single model. The model showed the naive cell differentiation into Th1, Th2, Th17 and Treg subtypes. The analysis predicted an unexpected plasticity behaviour of the canonical cell types as well as the potential of regulatory T cells to differentiate into Th1 or Th2 subtypes.

Another BN modelled the plasticity of *CD4 + T cell* differentiation [106] and showed that it is controlled by the dose and composition of cytokines. The model explained the T cell fate by defining 500 external conditions and considering all possible endogenous interactions. These interventions were perturbed to control the dynamics of the model from undesired to desired phenotypes. The model reproduced known synergistic actions of feedback loops on IL-12R expression and confirmed results from other studies [107], [108], showing that the balance between i-Treg and Th17 was regulated by IL-6. Furthermore, the model predicted a complex phenotype (Th1-Th2) after activation of Tbet and GATA3 transcription factors under the similar environmental conditions proposed by an in vivo study [109].

Integrating different layers of biological data allowed for understanding the heterogeneity of multifactorial diseases and for reducing the possibility of false positive results. A Boolean model of an integrative network was used to analyse the regulation of key transcription factors (TFs) in Rheumatoid Arthritis (RA) and derive patient-specific models to understand the disease complexity and the response to treatment [110]. The model highlighted the impact of TGFB1, IL6, and TNF in response to the anti-TNF drugs on the model outputs. The analysis showed that TFs are master regulators- the activation of IL6 and/or TGFB1 positively regulates TFs expression, even with deactivation of TNF cascade. Blocking IL6 and TGFB1, and TNF cascades deactivates TFs expression. Further, the MAPK molecules depend on the activation of IL6 and TGFB1 and do not be affected by TNF deactivation.

### 4.2 Modelling of cancer growth signalling and apoptosis

In cancer, targeted therapies inhibit driver molecules of tumorigenic pathways [112]. However, it is difficult to identify targets that have crucial functions in tumour progression because of complex interactions and feedback loops between implicated molecules. Moreover, monotherapies were found to be additive in their actions because tumours are highly complex and evolve continuously [30]. Therefore, they had limited efficacy and needed many clinical trials. Perturbation analysis may help to understand this complexity, proposing the interventions between molecular targets and predicting their possible synergistic action. The BNs of gastric cancer used this analysis on seven known inhibitors that target the gastric cancer pathways [97]. All possible combinations were calculated then simulated *in silico* to identify new synergistic targets, which were then experimentally validated. In another work, PBN allowed associating the activity of the pathological phenotype to the perturbation probability of its regulators. Under a given perturbation, the model tested the possible synergistic perturbations to decrease the activity of the phenotype [113].

Boolean modelling was proposed to simplify the complex interactions and their downstream signals. The molecular intervention analysis showed that the combinatory inhibition of oncogenic molecules e.g. PDK1, AKT, and MDM2 or the activation of P53, RB and CDH1 reduces the proliferation and increases quiescent phenotypes since the targeted drug associations blocked cancer pathways at different regions [112].

Signalling networks in cancer are complex cascades and their pathological rewiring may alter cellular proliferation, migration and apoptosis resistance [114], and BNs can help to understand this complicated rewiring [111], [115]. A Boolean model was constructed combining the main cancer pathways such as *RTKs, WNT/WNT/β-catenin*, *TGF-β/Smads, Rb, HIF-1, p53, PI3K/AKT signalling* pathways [114]. Identified attractors were associated with apoptosis, proliferation, and quiescent phenotypes in response to environmental conditions. The model revealed that growth factor signalling significantly increased the proliferation and quiescent phenotypes but decreased the apoptosis. The similar result was proposed by another model [116] which combined the intrinsic and extrinsic pro-apoptotic pathways with the growth factor signalling.

In another study, a BN describing the *PI3K/AKT1 signalling* pathway showed increased tissue proliferation and cell invasion phenotypes [115]. In particular, the oscillations of PI3K protein expression were studied by simulating its different activity levels at different cellular stages. Using different updating schemes can be more appropriate in specific settings and this is an example that illustrates it – While applying the synchronous updating scheme, the inhibition effect of PI3K induced four phenotypes including G2 arrest, mitotic catastrophe, and aberrant and normal anaphase. However, the asynchronous scheme showed that the previous four phenotypes didn’t occur at the same time, and they are not synergistic in signal transduction because the asynchronous scheme updates the biomolecules at different time intervals. Therefore, depending on the biological process and the knowledge about the real biological time, we get to decide which updating schemes make more sense to achieve a desired output.

Logical models of cancer are usually generic because they use heterogeneous data and require clinical data to calibrate them. To generate precise BNs, a PROFILE framework [114] was proposed, integrating the mechanistic insights of logical modelling with multi-omics data. The PROFILE framework combined mutations and expression data (METABRIC [117], TCGAdataset https://www.cancer.gov/tcga) with the cancer BN to simulate different cases and compare the model outputs. After data binarization, the activity of the nodes and the transition rates were modified based on specific cases. Stochastic simulations were performed using MaBoSS [91] for a semi-quantitative analysis of model perturbations. This approach was used in another study to investigate BRAF inhibition in melanoma and colorectal cancer which have significant variations despite the similar omics profiles [100]. The model was able to differentiate between the two cancers based on different datasets. This approach extends the previous works using the dynamic data [118] and the same pathways [119] to personalise the signalling behaviours in response to treatments.

Recently, researchers tested the PROFILE framework on a prostate model and infer patient-specific treatments [120]. The model of prostate cancer includes major deregulated signalling pathways integrated with mutation and RNAseq data. The biomolecules are fixed to zero/one according to the type of the mutations. For the continuous RNAseq data, the expression levels are translated as a modulation of a signal to the initial conditions to influence the probability of transitions. The analysis highlights that apoptosis is activated by Caspase 8/9, while proliferation is activated by cyclins D/B. Further, several readouts of cancer hallmarks (phenotypic outputs) were detected such as metastasis and DNA repair. The analysis identifies a list of drug combinations that reduce the proliferation phenotype or increase the apoptosis. The researchers use Boolean simulations to grade the effect of the combined drugs on patient-specific phenotypes, comparing the effects of treatments on each patient to suggest suitable treatment.

## 5 Perspectives

Computational models can improve understanding of complex disease mechanisms and help to develop treatment strategies applicable in the clinical settings. These applications need to be validated by closer integration with clinical research. To this end, modelling results and predictions need to be presented side by side with their uncertainties and biases. Despite successful applications of Boolean models in disease mechanism predictions and therapy (Table 2), scientists need to work on challenges of data integration, model building, precision, and standardisation.

We believe that working collectively toward solving these challenges will increase the development of decision-making pipelines using Boolean models in the future. Currently, clinical evaluation of Boolean models focuses on proposing personalised treatments. Further systematic approaches are required to study modes of action and doses besides studying the combinatorial effect on a single therapeutic target. Such approaches need to be tested and validated in vitro and on large cohorts, to better understand response and resistance to treatment, on a patient level.

The examples discussed in Section 4 show the ability of logical models to represent the dynamics of complex mechanisms. Still, more systematic approaches, including model curation, annotation and referencing in standardised formats, are needed to advance their application. Moreover, the application of Boolean models requires mathematical and bioinformatics expertise and can be made more understandable and reproducible by following established protocols [121]. Important parts of such protocols, to be defined before running simulations and experimental validations, should involve identifying the scope of the model, a choice of a justified modelling approach, and the strategy to reproduce the known behaviours.

It is important to keep the reproducibility of the generated models. This can be achieved using standardised formats (e.g., SBML packages) which facilitate the development of logical modelling pipelines (e.g., ColoMoTo notebook). Repositories like GINsim and CellCollective allow to construct, annotate, and share models. Still, reproducibility is a challenge and further integration of bioinformatic repositories with logical modelling, similarly to the BioModels platform (https://www.ebi.ac.uk/biomodels/) which already supports logical models [5]. However, a wide support to logical models requires involvement of communities Computational Modelling in Biology Network (COMBINE), the Simulation Experiment Description Markup Language (SED-ML) and SBML to advance the standards they develop. An example is the initiative of the ColoMoTo community and the Computational Modelling of Biological Systems (SysMod) community [122] to develop best practices for the curation and annotation of the logical models in biology [123]. Another example is the reproducibility scorecard, a list of eight questions to evaluate the reusability and reproducibility of the systems biology models [124]. In essence, a minimum information needs to be defined for the annotation of the logical models and systematically applied to enable their storage and reuse [123]. Automatic approaches of model annotation, quality assessment and curation will be crucial for this task.

## 6 Conclusions

Boolean modelling is a powerful and promising formalism to analyse a range of dynamic properties of the biological systems and disease mechanisms. It allows the use of many existing formats, including SBML-qual, SIF and GNA, offering the interoperability and the annotation of the created model. Boolean models need less parametrization than the quantitative models, making them a helpful approach to analyse less explored mechanisms. However, insufficient details in model construction may lead to inaccurate predictions. To avoid this, modellers can perform exploratory investigation, gathering the associated information of the model from literature and data resources. The missing details can be inferred by omics data integration that identifies the missing components and optimises the model accuracy. Another challenge is the model scale - complex models are harder to analyse and makes the attractor reachability very difficult [125], [126], [127]. This challenge motivates the scientists to propose different approaches to control complex models by reduction techniques.

Accurate and computable models improve the efficiency of simulations and the resulting analysis of their controllability. This makes Boolean models better suited for application in the areas of complex diseases such as cancer and immune cell differentiation. Therefore, it is crucial to emphasise the model quality in the construction and the analysis step. In parallel, the maintenance of the model repositories and sharing the models in easily interoperable formats are also important to improve their reproducibility. Further, the modelling community should cope with the advances in the experimental workflows and the new research findings by setting the best practices for the modelling process. This can be achieved by improving the existing models and trying to develop dedicated approaches to update the models automatically. Such reproducible models, further refined by omics data integration, will help to analyse the heterogeneity of complex diseases, simulate personalised responses to perturbations, and identify personalised treatments.

## CRediT authorship contribution statement

**Ahmed Abdelmonem Hemedan:** Investigation, Writing – original draft. **Anna Niarakis:** Conceptualization, Writing – review & editing. **Reinhard Schneider:** Supervision, Writing – review & editing. **Marek Ostaszewski:** Conceptualization, Writing – review & editing, Supervision.

## Declaration of Competing Interest

The authors declare that they have no known competing financial interests or personal relationships that could have appeared to influence the work reported in this paper.

## References

[1] del Valle E.P.G., García G.L., Santamaría L.P., Zanin M., Ruiz E.M., Rodríguez-González A. (2019). Disease networks and their contribution to disease understanding and drug repurposing: Evolution of the concept, techniques and data sources. BioRxiv.

[2] Ogata H., Goto S., Sato K., Fujibuchi W., Bono H., Kanehisa M. (1999). KEGG: Kyoto encyclopedia of genes and genomes. Nucl Acids Res.

[3] Wu M, Yang X, Chan C. A Dynamic Analysis of Insulin Signaling and Its Feedback Mechanisms: A Discrete Modeling Approach. 2009.

[4] Slenter D.N., Kutmon M., Hanspers K., Riutta A., Windsor J., Nunes N. (2018). WikiPathways: a multifaceted pathway database bridging metabolomics to other omics research. Nucl Acids Res.

[5] Malik-Sheriff R.S., Glont M., Nguyen T.V.N., Tiwari K., Roberts M.G., Xavier A. (2020). BioModels—15 years of sharing computational models in life science. Nucl Acids Res.

[6] Kazantsev F., Akberdin I., Lashin S., Ree N., Timonov V., Ratushny A. (2017). MAMMOTh: A new database for curated mathematical models of biomolecular systems. J Bioinform Comput Biol.

[7] Helikar T., Kowal B., McClenathan S., Bruckner M., Rowley T., Madrahimov A. (2012). The Cell Collective: toward an open and collaborative approach to systems biology. BMC Syst Biol.

[8] Naldi A, Hernandez C, Abou-Jaoudé W, Monteiro PT, Chaouiya C, Thieffry D. Logical Modeling and Analysis of Cellular Regulatory Networks With GINsim 3.0. Front Physiol 2018;9. doi: 10.3389/fphys.2018.00646. 10.3389/fphys.2018.00646 PMC6018412 29971008

[9] Wittig U., Kania R., Golebiewski M., Rey M., Shi L., Jong L. (2012). SABIO-RK–database for biochemical reaction kinetics. Nucl Acids Res.

[10] Mazein A., Ostaszewski M., Kuperstein I., Watterson S., Le Novère N., Lefaudeux D. (2018). Systems medicine disease maps: community-driven comprehensive representation of disease mechanisms. npj Syst Biol Appl.

[11] Funahashi A, Morohashi M, Matsuoka Y, Jouraku A, Kitano H. CellDesigner: A Graphical Biological Network Editor and Workbench Interfacing Simulator. In: Choi S, editor. Introd. Syst. Biol., Totowa, NJ: Humana Press; 2007, p. 422–34. doi: 10.1007/978-1-59745-531-2_21.

[12] Balci H., Siper M.C., Saleh N., Safarli I., Roy L., Kilicarslan M. (2020). Newt: a comprehensive web-based tool for viewing, constructing and analyzing biological maps. Bioinformatics.

[13] Wiese R, Eiglsperger M, Kaufmann M. yFiles — Visualization and Automatic Layout of Graphs. In: Jünger M, Mutzel P, editors. Graph Draw. Softw., Berlin, Heidelberg: Springer; 2004, p. 173–91. doi: 10.1007/978-3-642-18638-7_8.

[14] Kuperstein I., Cohen D.P., Pook S., Viara E., Calzone L., Barillot E. (2013). NaviCell: a web-based environment for navigation, curation and maintenance of large molecular interaction maps. BMC Syst Biol.

[15] Gawron P., Ostaszewski M., Satagopam V., Gebel S., Mazein A., Kuzma M. (2016). MINERVA-a platform for visualization and curation of molecular interaction networks. npj Syst Biol Appl.

[16] Aghamiri SS, Singh V, Naldi A, Helikar T, Soliman S, Niarakis A. Automated inference of Boolean models from molecular interaction maps using CaSQ. Bioinforma Oxf Engl 2020. https://doi.org/10/ghffmq. 10.1093/bioinformatics/btaa484 PMC7575051 32403123

[17] Silk D., Kirk P.D.W., Barnes C.P., Toni T., Stumpf M.P.H. (2014). Model selection in systems biology depends on experimental design. PLoS Comput Biol.

[18] Dubrova E. Random Multiple-Valued Networks: Theory and Applications. 36th Int. Symp. Mult.-Valued Log. ISMVL06, 2006, p. 27–27. doi: 10.1109/ISMVL.2006.36.

[19] van der Aalst WMP. Petri Nets. In: LIU L, ÖZSU MT, editors. Encycl. Database Syst., Boston, MA: Springer US; 2009, p. 2103–8. doi: 10.1007/978-0-387-39940-9_817.

[20] Walter W. Ordinary Differential Equations. New York: Springer-Verlag; 1998. doi: 10.1007/978-1-4612-0601-9.

[21] Ilea M., Turnea M., Rotariu M. (2012). Ordinary differential equations with applications in molecular biology. Rev Med Chir Soc Med Nat Iasi.

[22] Aldana M., Balleza E., Kauffman S., Resendiz O. (2007). Robustness and evolvability in genetic regulatory networks. J Theor Biol.

[23] Schlitt T., Brazma A. (2007). Current approaches to gene regulatory network modelling. BMC Bioinf.

[24] Maldonado E.M., Leoncikas V., Fisher C.P., Moore J.B., Plant N.J., Kierzek A.M. (2017). Integration of Genome Scale Metabolic Networks and Gene Regulation of Metabolic Enzymes With Physiologically Based Pharmacokinetics. CPT Pharmacomet Syst Pharmacol.

[25] Karr J.R., Sanghvi J.C., Macklin D.N., Gutschow M.V., Jacobs J.M., Bolival B. (2012). A whole-cell computational model predicts phenotype from genotype. Cell.

[26] Covert M.W., Xiao N., Chen T.J., Karr J.R. (2008). Integrating metabolic, transcriptional regulatory and signal transduction models in Escherichia coli. Bioinforma Oxf Engl.

[27] Chandrasekaran S., Price N.D. (2010). Probabilistic integrative modeling of genome-scale metabolic and regulatory networks in Escherichia coli and Mycobacterium tuberculosis. Proc Natl Acad Sci U S A.

[28] Albert R., Thakar J. (2014). Boolean modeling: a logic-based dynamic approach for understanding signaling and regulatory networks and for making useful predictions. Wiley Interdiscip Rev Syst Biol Med.

[29] Terfve C., Cokelaer T., Henriques D., MacNamara A., Goncalves E., Morris M.K. (2012). CellNOptR: a flexible toolkit to train protein signaling networks to data using multiple logic formalisms. BMC Syst Biol.

[30] Eduati F., Jaaks P., Wappler J., Cramer T., Merten C.A., Garnett M.J. (2020). Patient-specific logic models of signaling pathways from screenings on cancer biopsies to prioritize personalized combination therapies. Mol Syst Biol.

[31] Bloomingdale P., Nguyen V.A., Niu J., Mager D.E. (2018). Boolean network modeling in systems pharmacology. J Pharmacokinet Pharmacodyn.

[32] Tran V., McCall M.N., McMurray H.R., Almudevar A. (2013). On the underlying assumptions of threshold Boolean networks as a model for genetic regulatory network behavior. Front Genet.

[33] Albert I., Thakar J., Li S., Zhang R., Albert R. (2008). Boolean network simulations for life scientists. Source Code Biol Med.

[34] Hall B.A., Niarakis A. (2021). Data integration in logic-based models of biological mechanisms. Curr Opin Syst Biol.

[35] Schwab J.D., Kühlwein S.D., Ikonomi N., Kühl M., Kestler H.A. (2020). Concepts in Boolean network modeling: What do they all mean?. Comput Struct Biotechnol J.

[36] Tang Y.A., Pichler K., Füllgrabe A., Lomax J., Malone J., Munoz-Torres M.C. (2019). Ten quick tips for biocuration. PLoS Comput Biol.

[37] Varusai T.M., Jupe S., Sevilla C., Matthews L., Gillespie M., Stein L. (2021). Using Reactome to build an autophagy mechanism knowledgebase. Autophagy.

[38] Müssel C., Schmid F., Blätte T.J., Hopfensitz M., Lausser L., Kestler H.A. (2016). BiTrinA—multiscale binarization and trinarization with quality analysis. Bioinformatics.

[39] Ostrowski M., Paulevé L., Schaub T., Siegel A., Guziolowski C. (2016). Boolean Network Identification from Perturbation Time Series Data combining Dynamics Abstraction and Logic Programming. BioSystems.

[40] Haury A.-C., Mordelet F., Vera-Licona P., Vert J.-P. (2012). TIGRESS: Trustful Inference of Gene REgulation using Stability Selection. BMC Syst Biol.

[41] Aghamiri S.S., Delaplace F. (2021). TaBooN Boolean network synthesis based on Tabu Search. IEEE/ACM Trans Comput Biol Bioinform.

[42] Shmulevich I., Lähdesmäki H., Dougherty E.R., Astola J., Zhang W. (2003). The role of certain Post classes in Boolean network models of genetic networks. Proc Natl Acad Sci U S A.

[43] Martin S., Zhang Z., Martino A., Faulon J.-L. (2007). Boolean dynamics of genetic regulatory networks inferred from microarray time series data. Bioinforma Oxf Engl.

[44] Liang S., Fuhrman S., Somogyi R. (1998). Reveal, a general reverse engineering algorithm for inference of genetic network architectures. Pac Symp Biocomput Pac Symp Biocomput.

[45] Berestovsky N., Nakhleh L. (2013). An evaluation of methods for inferring boolean networks from time-series data. PLoS ONE.

[46] Venkateswarlu C. (2021). A metaheuristic Tabu search optimization algorithm: applications to chemical and environmental processes. IntechOpen.

[47] Razzaq M., Paulevé L., Siegel A., Saez-Rodriguez J., Bourdon J., Guziolowski C. (2018). Computational discovery of dynamic cell line specific boolean networks from multiplex time-course data. PLoS Comput Biol.

[48] Videla S., Saez-Rodriguez J., Guziolowski C., Siegel A. (2017). caspo: a toolbox for automated reasoning on the response of logical signaling networks families. Bioinformatics.

[49] Thiele S. PyASP 1.4.1 - A convenience wrapper for the ASP tools gringo, gringo4 and clasp. Zenodo; 2015. https://doi.org/10.5281/zenodo.22968.

[50] Wang R.-S., Saadatpour A., Albert R. (2012). Boolean modeling in systems biology: an overview of methodology and applications. Phys Biol.

[51] Drossel B. Random Boolean Networks. Rev. Nonlinear Dyn. Complex., John Wiley & Sons, Ltd; 2008, p. 69–110. https://doi.org/10.1002/9783527626359.ch3.

[52] Kauffman S., Peterson C., Samuelsson B., Troein C. (2004). Genetic networks with canalyzing Boolean rules are always stable. Proc Natl Acad Sci.

[53] Bilke S., Sjunnesson F. (2002). Stability of the Kauffman model. Phys Rev E: Stat Nonlinear Soft Matter Phys.

[54] Zañudo JGT, Aldana M, Martínez-Mekler G. Boolean Threshold Networks: Virtues and Limitations for Biological Modeling. In: Niiranen S, Ribeiro A, editors. Inf. Process. Biol. Syst., Berlin, Heidelberg: Springer; 2011, p. 113–51. doi: 10.1007/978-3-642-19621-8_6.

[55] Irwin M, Wang Z. Dynamic Systems Modeling. Int. Encycl. Commun. Res. Methods, American Cancer Society; 2017, p. 1–12. doi: 10.1002/9781118901731.iecrm0074.

[56] Campbell C., Albert R. (2019). Edgetic perturbations to eliminate fixed-point attractors in Boolean regulatory networks. Chaos Interdiscip J Nonlinear Sci.

[57] Garg A., Di Cara A., Xenarios I., Mendoza L., De Micheli G. (2008). Synchronous versus asynchronous modeling of gene regulatory networks. Bioinformatics.

[58] Saadatpour A., Albert R., Reluga T. (2013). A Reduction Method for Boolean Network Models Proven to Conserve Attractors. SIAM J Appl Dyn Syst.

[59] Chaves M., Figueiredo D., Martins M.A. (2020). Boolean dynamics revisited through feedback interconnections. Nat Comput.

[60] Hopfensitz M., Mussel C., Wawra C., Maucher M., Kuhl M., Neumann H. (2012). Multiscale binarization of gene expression data for reconstructing Boolean networks. IEEE/ACM Trans Comput Biol Bioinform.

[61] Klemm K., Bornholdt S. (2005). Stable and unstable attractors in Boolean networks. Phys Rev E.

[62] Fauré A., Naldi A., Chaouiya C., Thieffry D. (2006). Dynamical analysis of a generic Boolean model for the control of the mammalian cell cycle. Bioinforma Oxf Engl.

[63] Shmulevich I., Kauffman S.A., Aldana M. (2005). Eukaryotic cells are dynamically ordered or critical but not chaotic. Proc Natl Acad Sci.

[64] Serra R., Villani M., Graudenzi A., Kauffman S.A. (2007). Why a simple model of genetic regulatory networks describes the distribution of avalanches in gene expression data. J Theor Biol.

[65] Müssel C., Hopfensitz M., Kestler H.A. (2010). BoolNet–an R package for generation, reconstruction and analysis of Boolean networks. Bioinforma Oxf Engl.

[66] Biere A. (2008). PicoSAT Essentials. J Satisf Boolean Model Comput.

[67] Veliz-Cuba A. (2011). Reduction of Boolean network models. J Theor Biol.

[68] Veliz-Cuba A., Aguilar B., Hinkelmann F., Laubenbacher R. (2014). Steady state analysis of Boolean molecular network models via model reduction and computational algebra. BMC Bioinf.

[69] Moradi M., Goliaei S., Foroughmand-Araabi M.-H. (2019). A Boolean network control algorithm guided by forward dynamic programming. PLoS ONE.

[70] Paulevé L., Kolčák J., Chatain T., Haar S. (2020). Reconciling qualitative, abstract, and scalable modeling of biological networks. Nat Commun.

[71] Paulevé L., Richard A. (2012). Static analysis of Boolean networks based on interaction graphs: a survey. Electron Notes Theor Comput Sci.

[72] Wolf I.R., Simões R.P., Valente G.T. (2021). Three topological features of regulatory networks control life-essential and specialized subsystems. Sci Rep.

[73] Xiao Y., Dougherty E.R. (2007). The impact of function perturbations in Boolean networks. Bioinformatics.

[74] Trinh H.-C., Kwon Y.-K. (2019). RMut: R package for a Boolean sensitivity analysis against various types of mutations. PLoS ONE.

[75] Kobayashi K., Hiraishi K. (2017). Optimization-based approaches to control of probabilistic Boolean networks. Algorithms.

[76] Poret A., Guziolowski C. (2018). Therapeutic target discovery using Boolean network attractors: improvements of kali. R Soc Open Sci.

[77] Hu M., Shen L., Zan X., Shang X., Liu W. (2016). An efficient algorithm to identify the optimal one-bit perturbation based on the basin-of-state size of Boolean networks. Sci Rep.

[78] Shannon P., Markiel A., Ozier O., Baliga N.S., Wang J.T., Ramage D. (2003). Cytoscape: a software environment for integrated models of biomolecular interaction networks. Genome Res.

[79] Türei D., Korcsmáros T., Saez-Rodriguez J. (2016). OmniPath: guidelines and gateway for literature-curated signaling pathway resources. Nat Methods.

[80] Licata L, Lo Surdo P, Iannuccelli M, Palma A, Micarelli E, Perfetto L, et al. SIGNOR 2.0, the SIGnaling Network Open Resource 2.0: 2019 update. Nucl Acids Res 2020;48:D504–10. doi: 10.1093/nar/gkz949. 10.1093/nar/gkz949 PMC7145695 31665520

[81] Karanam A, He D, Hsu P-K, Schulze S, Dubeaux G, Karmakar R, et al. BoolSim, a Graphical Interface for Open Access Boolean Network Simulations and Use in Guard Cell CO2 Signaling. BioRxiv 2021:2021.03.05.434139. doi: 10.1101/2021.03.05.434139. 10.1093/plphys/kiab344 PMC8644243 34618035

[82] Di Cara A., Garg A., De Micheli G., Xenarios I., Mendoza L. (2007). Dynamic simulation of regulatory networks using SQUAD. BMC Bioinf.

[83] Batt G, Besson B, Ciron P-E, de Jong H, Dumas E, Geiselmann J, et al. Genetic Network Analyzer: A Tool for the Qualitative Modeling and Simulation of Bacterial Regulatory Networks. In: van Helden J, Toussaint A, Thieffry D, editors. Bact. Mol. Netw. Methods Protoc., New York, NY: Springer; 2012, p. 439–62. https://doi.org/10.1007/978-1-61779-361-5_22. 10.1007/978-1-61779-361-5_22 22144166

[84] Naldi A, Monteiro PT, Müssel C, Consortium for Logical Models and Tools, Kestler HA, Thieffry D, et al. Cooperative development of logical modelling standards and tools with CoLoMoTo. Bioinforma Oxf Engl 2015;31:1154–9. doi: 10.1093/bioinformatics/btv013. 10.1093/bioinformatics/btv013 25619997

[85] Hucka M, Bergmann F, Hoops S, Keating SM, Novère NL, Myers CJ, et al. Systems Biology Markup Language (SBML) Level 3Core 2019:182.

[86] Chaouiya C., Bérenguier D., Keating S.M., Naldi A., van Iersel M.P., Rodriguez N. (2013). SBML qualitative models: a model representation format and infrastructure to foster interactions between qualitative modelling formalisms and tools. BMC Syst Biol.

[87] Büchel F., Rodriguez N., Swainston N., Wrzodek C., Czauderna T., Keller R. (2013). Path2Models: large-scale generation of computational models from biochemical pathway maps. BMC Syst Biol.

[88] Le D.-H., Kwon Y.-K. (2011). NetDS: a Cytoscape plugin to analyze the robustness of dynamics and feedforward/feedback loop structures of biological networks. Bioinformatics.

[89] Su C., Pang J. (2021). CABEAN: a software for the control of asynchronous Boolean networks. Bioinformatics.

[90] Hinkelmann F., Brandon M., Guang B., McNeill R., Blekherman G., Veliz-Cuba A. (2011). ADAM: analysis of discrete models of biological systems using computer algebra. BMC Bioinf.

[91] Stoll G., Caron B., Viara E., Dugourd A., Zinovyev A., Naldi A. (2017). MaBoSS 2.0: an environment for stochastic Boolean modeling. Bioinforma Oxf Engl.

[92] Correia R.B., Gates A.J., Wang X., Rocha L.M. (2018). CANA: a Python package for quantifying control and canalization in Boolean networks. Front Physiol.

[93] Mizera A, Pang J, Yuan Q. ASSA-PBN: An Approximate Steady-State Analyser of Probabilistic Boolean Networks. In: Finkbeiner B, Pu G, Zhang L, editors. Autom. Technol. Verification Anal., Cham: Springer International Publishing; 2015, p. 214–20. doi: 10.1007/978-3-319-24953-7_16.

[94] Benque D, Bourton S, Cockerton C, Cook B, Fisher J, Ishtiaq S, et al. Bma: Visual Tool for Modeling and Analyzing Biological Networks. In: Madhusudan P, Seshia SA, editors. Comput. Aided Verification, vol. 7358, Berlin, Heidelberg: Springer Berlin Heidelberg; 2012, p. 686–92. doi: 10.1007/978-3-642-31424-7_50.

[95] Elmeligy Abdelhamid S.H., Kuhlman C.J., Marathe M.V., Mortveit H.S., Ravi S.S. (2015). GDSCalc: a web-based application for evaluating discrete graph dynamical systems. PLoS ONE.

[96] Paroni A., Graudenzi A., Caravagna G., Damiani C., Mauri G., Antoniotti M. (2016). CABeRNET: a Cytoscape app for augmented Boolean models of gene regulatory NETworks. BMC Bioinf..

[97] Flobak Å., Baudot A., Remy E., Thommesen L., Thieffry D., Kuiper M. (2015). Discovery of Drug Synergies in Gastric Cancer Cells Predicted by Logical Modeling. PLoS Comput. Biol..

[98] Bruner A., Sharan R. (2019). A robustness analysis of dynamic Boolean models of cellular circuits. J Comput Biol.

[99] Kwon Y.-K., Kim J., Cho K.-H. (2015). Dynamical robustness against multiple mutations in signaling networks. IEEEACM Trans Comput Biol Bioinforma IEEE ACM.

[100] Béal J., Pantolini L., Noël V., Barillot E., Calzone L. (2021). Personalized logical models to investigate cancer response to BRAF treatments in melanomas and colorectal cancers. PLoS Comput Biol.

[101] Siegle L., Schwab J.D., Kühlwein S.D., Lausser L., Tümpel S., Pfister A.S. (2018). A Boolean network of the crosstalk between IGF and Wnt signaling in aging satellite cells. PLoS ONE.

[102] Campbell C., Albert R. (2014). Stabilization of perturbed Boolean network attractors through compensatory interactions. BMC Syst Biol.

[103] Gjerga E, Trairatphisan P, Gabor A, Koch H, Chevalier C, Ceccarelli F, et al. Converting networks to predictive logic models from perturbation signalling data with CellNOpt. BioRxiv 2020:2020.03.04.976852. doi: 10.1101/2020.03.04.976852. 10.1093/bioinformatics/btaa561 PMC7575044 32516357

[104] Saez-Rodriguez J., Simeoni L., Lindquist J.A., Hemenway R., Bommhardt U., Arndt B. (2007). A logical model provides insights into T cell receptor signaling. PLoS Comput Biol.

[105] Naldi A., Carneiro J., Chaouiya C., Thieffry D. (2010). Diversity and plasticity of Th cell types predicted from regulatory network modelling. PLoS Comput Biol.

[106] Puniya B.L., Todd R.G., Mohammed A., Brown D.M., Barberis M., Helikar T. (2018). A mechanistic computational model reveals that plasticity of CD4+ T cell differentiation is a function of cytokine composition and dosage. Front Physiol.

[107] Yang X.O., Nurieva R., Martinez G.J., Kang H.S., Chung Y., Pappu B.P. (2008). Molecular antagonism and plasticity of regulatory and inflammatory T cell programs. Immunity.

[108] Kimura A., Kishimoto T. (2010). IL-6: regulator of Treg/Th17 balance. Eur J Immunol.

[109] Peine M., Rausch S., Helmstetter C., Fröhlich A., Hegazy A.N., Kühl A.A. (2013). Stable T-bet+GATA-3+ Th1/Th2 Hybrid Cells Arise In Vivo, Can Develop Directly from Naive Precursors, and Limit Immunopathologic Inflammation. PLoS Biol.

[110] Miagoux Q., Singh V., de Mézquita D., Chaudru V., Elati M., Petit-Teixeira E. (2021). Inference of an integrative, executable network for rheumatoid arthritis combining data-driven machine learning approaches and a state-of-the-art mechanistic disease map. J Pers Med.

[111] Fumiã H.F., Martins M.L. (2013). Boolean network model for cancer pathways: predicting carcinogenesis and targeted therapy outcomes. PLoS ONE.

[112] Luo J., Solimini N.L., Elledge S.J. (2009). Principles of cancer therapy: oncogene and non-oncogene addiction. Cell.

[113] Irurzun-Arana I., Pastor J.M., Trocóniz I.F., Gómez-Mantilla J.D. (2017). Advanced Boolean modeling of biological networks applied to systems pharmacology. Bioinformatics.

[114] Béal J., Montagud A., Traynard P., Barillot E., Calzone L. (2019). Personalization of logical models with multi-omics data allows clinical stratification of patients. Front Physiol.

[115] Sizek H., Hamel A., Deritei D., Campbell S., Regan E.R. (2019). Boolean model of growth signaling, cell cycle and apoptosis predicts the molecular mechanism of aberrant cell cycle progression driven by hyperactive PI3K. PLoS Comput Biol.

[116] Z M, H L. Boolean network-based analysis of the apoptosis network: irreversible apoptosis and stable surviving. J Theor Biol 2009;259:760–9. https://doi.org/10.1016/j.jtbi.2009.04.024. 10.1016/j.jtbi.2009.04.024 19422837

[117] C C, Sp S, Sf C, G T, Om R, Mj D, et al. The genomic and transcriptomic architecture of 2,000 breast tumours reveals novel subgroups. Nature 2012;486:346–52. doi: 10.1038/nature10983. 10.1038/nature10983 PMC3440846 22522925

[118] Saez-Rodriguez J., Blüthgen N. (2020). Personalized signaling models for personalized treatments. Mol Syst Biol.

[119] Klinger B., Sieber A., Fritsche-Guenther R., Witzel F., Berry L., Schumacher D. (2013). Network quantification of EGFR signaling unveils potential for targeted combination therapy. Mol Syst Biol.

[120] Montagud A., Béal J., Tobalina L., Traynard P., Subramanian V., Szalai B. (2022). Patient-specific Boolean models of signalling networks guide personalised treatments. ELife.

[121] Niarakis A., Helikar T. (2020). A practical guide to mechanistic systems modeling in biology using a logic-based approach. Brief Bioinform.

[122] Dräger A., Helikar T., Barberis M., Birtwistle M., Calzone L., Chaouiya C. (2021). SysMod: the ISCB community for data-driven computational modelling and multi-scale analysis of biological systems. Bioinformatics.

[123] Niarakis A., Kuiper M., Ostaszewski M., Malik Sheriff R.S., Casals-Casas C., Thieffry D. (2021). Setting the basis of best practices and standards for curation and annotation of logical models in biology—highlights of the [BC]2 2019 CoLoMoTo/SysMod Workshop. Brief Bioinform.

[124] Tiwari K., Kananathan S., Roberts M.G., Meyer J.P., Sharif Shohan M.U., Xavier A. (2021). Reproducibility in systems biology modelling. Mol Syst Biol.

[125] Garg A., Mohanram K., Di Cara A., De Micheli G., Xenarios I. (2009). Modeling stochasticity and robustness in gene regulatory networks. Bioinforma Oxf Engl.

[126] Murrugarra D., Veliz-Cuba A., Aguilar B., Arat S., Laubenbacher R. (2012). Modeling stochasticity and variability in gene regulatory networks. EURASIP J Bioinforma Syst Biol.

[127] Koltai M., Noel V., Zinovyev A., Calzone L., Barillot E. (2020). Exact solving and sensitivity analysis of stochastic continuous time Boolean models. BMC Bioinf.

